# Evaluating the Association Between Neutrophil Gelatinase-Associated Lipocalin Levels and Periodontal Health Status in Patients With Chronic Kidney Disease

**DOI:** 10.1155/mi/4114758

**Published:** 2025-09-24

**Authors:** Ahmed Mutar, Maha Sh Mahmood

**Affiliations:** Department of Periodontics, College of Dentistry, University of Baghdad, Baghdad, Iraq

**Keywords:** chronic kidney disease (CKD), NGAL, periodontal health

## Abstract

**Background:** Chronic kidney disease (CKD) is one of the significant public health problems that is characterized by structural and functional changes due to various causes. Periodontal disease has risen as a nontraditional risk factor for CKD since it is considered a source of inflammatory products in systemic disease.

**Objective:** The objective of the study was to investigate the association between serum and salivary levels of neutrophil gelatinase-associated lipocalin (NGAL) and the periodontal health status of patients with CKD.

**Methodology:** A cross-sectional study was carried out to achieve the goal. It involved 150 adult patients with CKD, with and without hemodialysis (HD). Assessment of the periodontal health status was done through the measurement of periodontal parameters. Moreover, samples of saliva and serum were collected from each patient and examined for NGAL levels using an enzyme-linked immunosorbent test.

**Results:** The study findings illustrated a significant elevation in the mean concentration of NGAL in saliva in comparison to serum in both groups (saliva 540.554 ± 100.595 ng/mL, 458.134 ± 99.450 ng/mL) versus serum (274.138 ± 70.18 ng/mL, 351.66 ± 99.451 ng/mL) (*p*  < 0.001). As well as significant elevation of the biomarker was also pronounced in the saliva of the CKD group not on HD in comparison with the HD group (540.554 ± 100.595 ng/mL) versus (458.134 ± 99.450 ng/mL), respectively, *p*-value < 0.001. Regarding periodontal health status, findings showed that from the total sample of patients comprising 150 patients, the majority of them had periodontitis (42%) in the non-HD group and (38.6%) in the HD group (total 80.6%) while the prevalence of gingivitis was (8%) in non-HD group and (11.3%) in HD group (total 19.3%). In addition, results revealed a significant association of serum and salivary levels of NGAL with patients who have CKD on HD. On the other hand, the association with periodontal parameters was only clarified with the salivary level of NGAL.

**Conclusion:** Elevated NGAL levels in saliva were significantly associated with CKD patients on HD as well as with clinical periodontal parameters.

## 1. Introduction

The primary kidney function is to sustain homeostasis by eliminating electrolytes, water, and metabolic waste products from the body. Chronic renal failure (CRF) is a gradual deterioration in kidney function that is linked to a decrease in the rate at which blood is filtered by the kidney's glomeruli. The cause is the gradual and long-term harm to the nephrons, which requires the use of additional ways to filter the blood outside of the kidneys, primarily through hemodialysis (HD) [1].

The kidneys play a crucial part in maintaining metabolic balance, as evidenced by the fact that kidney failure has been demonstrated to result in anemia, high blood pressure, nerve damage, thyroid malfunction, and decreased sexual desire [2].

The HD is a synthetic procedure utilized to eliminate nitrogenous waste and other hazardous metabolic byproducts from the bloodstream. It enhances the well-being of individuals with irreversible, severe kidney damage caused by glomerulonephritis, obstructive uropathy, unresolved acute tubular necrosis, interstitial nephritis, renal toxicity, nephrolithiasis, and systemic diseases associated with end-stage renal disease (ESRD) [3].

The lowered immunological responses and medication side effects that disguise infection symptoms make HD patients more vulnerable to infection-related mortality and morbidity [4]. Patients undergoing dialysis commonly exhibit a range of oral manifestations, such as mucosal lesions, oral infections, tooth anomalies, and bone lesions caused by secondary hyperparathyroidism. Other observed conditions include malocclusion, accelerated dental calculus formation, inflammation of the gingiva, mucosal pallor and lesions, an altered microbiological environment, tooth mobility, and a heightened susceptibility to tooth erosion [5–7]. Because oral diseases or infections could compromise the transplantation treatment, HD patients who are waiting for a kidney transplant must maintain good oral health [8].

Periodontal diseases refer to infectious and inflammatory conditions that affect the tissues supporting the teeth, including the alveolar bone and periodontal ligament. The primary clinical feature of these conditions is the detachment of the tooth from its supporting structures, leading to the formation of periodontal pockets and alterations in the density of the underlying alveolar bone [9, 10]. There is overwhelming evidence of an association between chronic kidney disease (CKD) and periodontitis [11], both are associated with an increase in oxidative stress and inflammatory mediators [12–14].

NGAL, which stands for neutrophil gelatinase-associated lipocalin, is also referred to as human neutrophil lipocalin or lipocalin 2. This protein was initially discovered in the secondary granules of human neutrophils and is characterized by a molecular weight of 25 kilodaltons (kDa) [15, 16].

When a bacterial infection occurs, NGAL levels in the bloodstream rise significantly, with its concentration in both serum and urine increasing dramatically within hours of inflammation. NGAL plays a crucial role in iron transport, helping to move iron to and from cells. In particular, it aids in delivering iron to kidney tubular cells and may be involved in the repair process during acute kidney injury (AKI) by promoting the differentiation of renal progenitor cells into epithelial tubules [17, 18].

NGAL, a biomarker of inflammation, plays a critical role in modulating host immune responses and iron homeostasis. Its elevated levels in periodontitis are associated with neutrophilic activity, metalloproteinase (MMP) release, and tissue destruction. NGAL contributes to periodontal disease through its role in inflammation, oxidative stress, interaction with matrix MMP, and systemic inflammation, particularly in CKD patients. These mechanisms collectively promote tissue destruction and alveolar bone loss, exacerbating periodontal disease [19].

Several studies have investigated NGAL levels in periodontal disease. For example, Youssef et al. [20] found that NGAL levels in the gingival crevicular fluid were significantly higher in periodontitis patients compared to healthy individuals, suggesting that NGAL could serve as a potential inflammatory biomarker for periodontal disease.

HD may influence NGAL levels due to increased systemic inflammation and oxidative stress, resulting in elevated NGAL in circulation and the secondary outcomes of this relationship on periodontal disease may include intensive periodontal tissue destruction, delayed healing, and exacerbation of periodontal inflammation in CKD patients [21].

The current study addresses a gap in understanding how systemic conditions like CKD alter NGAL levels and their association with the periodontal health status of CKD patients on HD.

However, there has been no research specifically measuring NGAL levels to evaluate the relationship between CKD patients on HD and their periodontal health status. Therefore, the current study aims to assess the periodontal health status of individuals with CKD and explore the relationship between periodontal status and salivary and serum levels of NGAL.

## 2. Materials and Methods

### 2.1. Study Design

The chosen design for this study to achieve its objectives was an observational cross-sectional study. This study was carried out from January to July 2023 at Al-Hussein Teaching Hospital inside the Al-Muthanna Directorate, including patients with CKD regardless of the cause of their illness. The individuals were separated into two distinct groups, with the first group comprising individuals with CKD undergoing HD. The second group comprised patients with chronic renal illnesses who were not undergoing HD. These individuals were matched with the first group based on their medical history, age, and sex.

The inclusion criteria for eligible participants in this study were individuals aged 18 years or older who had a minimum of 10 teeth [22]. Additionally, the patients needed to have a confirmed diagnosis of ESRD based on the clinical, biochemical, or ultrasound findings. They could either be receiving conservative care without dialysis treatment or undergoing HD two times a week for a minimum of 6 months and a maximum of 1 year and 3–4 h/per session. They had to possess the ability to provide consent as well as participate in an oral examination.

The exclusion criteria consisted of patients with serious medical conditions, such as malignancy, individuals who had received kidney transplants, patients who received periodontal therapy within the past 3 months, individuals who declined to participate in the research, pregnant or breastfeeding women, and those who tested positive for hepatitis (B or C) or human immunodeficiency virus (HIV).

### 2.2. Data Collection and Ethical Considerations

The researchers obtained ethical approval for the present work from the Research Ethical Committees of the College of Dentistry, University of Baghdad, and followed the guidelines of the Declaration of Helsinki and Tokyo for humans [23] with protocol number: 825,623 on July 01, 2023. Written informed consent was obtained from all participants before their enrollment.

### 2.3. Sample Size Calculation

For the calculation of the required sample size, the serum concentration of NGAL was used as a primary outcome. A pilot study used the first 10 samples obtained from each group. In total, enzyme-linked immunosorbent assay (ELISA) was used in the laboratory to examine the selected samples and the concentration of NGAL gained from a pilot study was used to determine the size of the sample [24]. With an allocation ratio of one-to-one, the sample size was rounded up to 75 for each group, even though the needed sample size for each study group was 65. This was done to allow for the possibility of a dropout rate of 10%.

The sampling method employed in this observational cross-sectional study is convenience sampling, a type of nonrandom sampling that selects readily available subjects. This approach is commonly used in medical research and involves recruiting clinical cases or participants who are accessible at a specific location, such as a hospital.

### 2.4. Collection of Serum Samples

All subjects had blood drawn between 9 and 11 AM. The patient arms' cubital fossa or double lumens were used to collect 3 cc of blood. Numbers were assigned to samples based on patient names and groups. In patients undergoing HD, the samples were obtained before giving the patient heparin and connecting them to dialysis. A cooling box was used to prevent the denaturation of proteins after collection. At 2–8°C, samples were centrifuged for 15 min at 3000 rpm. Afterwards, refrigerated in an aliquot at −20°C [25].

### 2.5. Collection of Salivary Samples

Following the guidelines of the University of Southern California/School of Dentistry [26, 27], participants were instructed to avoid eating, drinking, or performing oral hygiene activities for at least 30 min before saliva collection. Samples were collected using the passive drooling method with the head tilted forward, into sterile containers, following established protocols.

The protocol prioritized participant relaxation and mandated the use of sterilized water for oral rinsing before collection. Specimens tainted with blood were disposed of. The collected samples underwent centrifugation at a speed of 3000 revolutions per minute for 10 min. The resulting supernatant liquid portion was preserved at a temperature of −20˚C for subsequent analysis.

### 2.6. ELISA

The detection of NGAL in serum and salivary samples was done through the utilization of human ELISA quantitative. The reference range for NGAL levels was determined based on the manufacturer's instructions for the ELISA kit used in the study (Shanghai YL Biotech Co., Ltd., Catalog #YLA0724HU). The standard curve was established by running a series of known NGAL concentrations provided with the kit, ranging from 0 to 400 ng/mL (0, 10, 50, 100, 200, and 400 ng/mL).

Each standard concentration was run in duplicate, and the absorbance values were plotted against the concentrations to create the curve. A four-parameter logistic (4PL) regression model was used to fit the data, achieving a correlation coefficient (*R*^2^) of 0.99. The NGAL levels in the samples were then calculated by interpolating their absorbance values on the standard curve.

### 2.7. Periodontal Examination

The classification of periodontal disease was based on the 2017 World Workshop on the Classification of Periodontal and Peri-Implant Diseases and Conditions. Individuals were categorized according to their periodontal health status which was defined in terms of the following:

1. Clinically healthy gingiva on intact and reduced periodontium in which bleeding on probing (BOP) scores less than 10%, probing pocket depth (PPD) less than 3 mm.
2. Biofilm-induced gingivitis in which BOP is more than 10% with PPD equal to or less than 3 mm. The gingivitis patients either generalized gingivitis in which more than 30% of sites are affected or localized gingivitis in which less than 30% of sites are affected by the disease.
3. Periodontitis cases were diagnosed as having interdental CAL was detectable at ≥2 nonadjacent teeth, or buccal or oral CAL ≥3 mm with pocketing >3 mm is detectable at ≥2 teeth. The periodontitis patients either generalized periodontitis in which more than 30% of teeth are included in bone loss or localized periodontitis in which less than 30% of teeth are included in bone loss [28].

### 2.8. Calibration

It was determined that the examiner accuracy and repeatability of clinical periodontal measures (CAL, PPD, BOP, and plaque index [PLI]) were validated by the following:

#### 2.8.1. Interexaminer Calibration

A periodontal expert and the researcher analyzed the periodontal parameters (CAL, PPD, BOP, and PLI) for 10 subjects at the same time.

#### 2.8.2. Intraexaminer Calibration

The parameters of periodontal (CAL, PPD, BOP, and PLI) of 10 individuals were evaluated by the researcher. To promote intraexaminer calibration, the researcher allowed for a 2 h gap between each examination.

Using the kappa-coefficient assay, interexaminer and intraexaminer calibration for categorical variables (PI and BOP) was evaluated. The requisite threshold for determining a satisfactory level of agreement was a kappa value of at least 75%. Intraclass correlation coefficient (ICC) used to determine the level of agreement, for continuous variables (PPD and CAL) ranged from 0.758 to 0.950. There is a significant absolute agreement correlation between the two readings.

A calibrated examiner used a UNC-15 periodontal probe to assess BOP, PPD, and clinical attachment loss (CAL) at six sites per tooth for all teeth except wisdom teeth. The PLI recorded plaque on all teeth's mesial, distal, buccal, and lingual surfaces using the O'Leary index [29]. In the case of BOP, the periodontal probe was carefully placed into either the gingival sulcus or the periodontal pocket at six specific locations per tooth. If bleeding occurred within 10–30 s, it was given a score of 1, while nonbleeding surfaces were given a score of 0 [30, 31].

The PPD was measured from the gingival margin to the deepest probe penetration in the pocket or the sulcus. Like PPD, CAL, the distance from the cementoenamel junction (CEJ) to the pocket base, matches PPD when the gingival border aligns with the CEJ, is higher if apical, and less coronal.

### 2.9. Statistical Analysis

Data were analyzed using statistical software (SPSS version-22, Chicago, Illinois, United States of America and Graph pad Prism 9.5.1). The Shapiro–Wilk test was used to check the normality of distributions, and the Levene test for homogeneity of variances among groups.

Continuous data were summarized as means and standard deviations(SDs), while categorical data were presented as frequencies and percentages. Comparisons between groups were conducted using appropriate tests, such as *t*-tests, ANOVA, or nonparametric equivalents, depending on the data. Multivariate regression models were used to account for the biomarker-periodontal health status-CKD relationship [32]. A *p*-value of less than 0.05 was considered statistically significant.

## 3. Results

About 198 patients were examined and only 150 patients fit the inclusion criteria while 48 patients were excluded as they did not fill the needed criteria. The included patients were divided into two equal groups (75 patients in each group).

### 3.1. Periodontal Health Status and Demographic Data

The distribution of periodontal health status in detail was described in Table 1 and there was a significant increase in generalized gingivitis in chronic kidney patients on HD, while localized gingivitis predominates the patients without HD. Besides, periodontitis dominates the patients with CKD without statistical significance in patients with and without HD. Concerning the demographic data (Table 2) including age and gender the distribution of age between groups was statistically nonsignificant. While sex distribution, the sample was significantly composed of men affected with periodontitis in patients with CKD without HD. However, the distribution of subjects according to sex between groups was nonsignificant.

#### 3.1.1. Periodontal Parameters

Concerning the periodontal parameters the results in Table 3 have shown that all periodontal parameters were higher in generalized and localized periodontitis patients than generalized and localized gingivitis in both groups. Nevertheless, statistical analysis has illustrated there was a significant increase in the mean PLI% and mean BOP% among patients with localized gingivitis in patients without HD in comparison with another group. Subsequently, there was a significant increase in the mean PLI% among patients with gingivitis in patients with CKD without HD. Conversely, patients with CKD on HD with periodontitis presented a significant increase in the mean PPD in comparison with others.

#### 3.1.2. Biomarker Concentration

In Table 4, the mean concentration of NGAL in ng/mL for serum and saliva was presented. The concentration of biomarkers was significantly higher in saliva in comparison with serum. In detail, the concentration of NGAL in serum was significantly higher in patients with gingivitis precise with localized gingivitis in patients on HD in comparison with others. Whereas the concentration of NGAL in saliva was significantly higher in gingivitis and periodontitis in patients with CKD without HD as offered below.

#### 3.1.3. Regression Analysis for Biomarker

**Figure 1:**
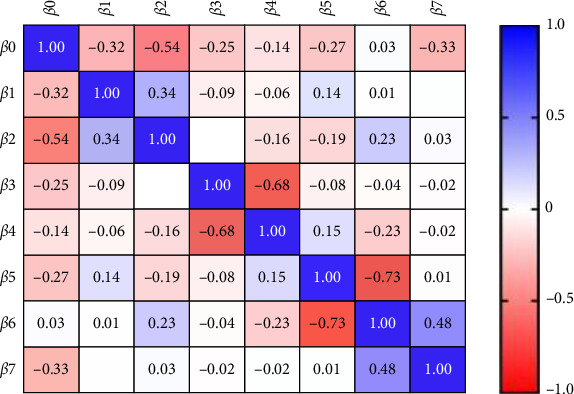
Parameter covariance in the first model of multiple linear regressions.

Multiple linear regression analysis was done in different models to evaluate the association of the biomarker with disease progression since CKD was treated as a categorical variable, considering patient on HD as 1 while without HD was reported as 0. Moreover periodontal health status was measured the same giving score 1 to periodontitis while score 0 to gingivitis. The first model was illustrated in Table 5 and Figure 1 where NGAL in serum was considered the dependent variable the result showed that significant impact of CKD on HD on the serum level of NGAL, while the other factor showed a nonsignificant result.

**Figure 2:**
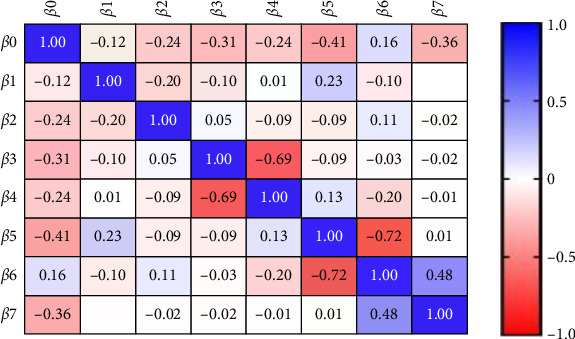
Parameter covariance in the second model of multiple linear regression.

Furthermore, considering the concentration of NGAL in saliva as a dependent variable the result revealed a significant association of biomarkers with patients on HD as well as with PPD and CAL as illustrated in Table 6 and Figure 2.

**Figure 3:**
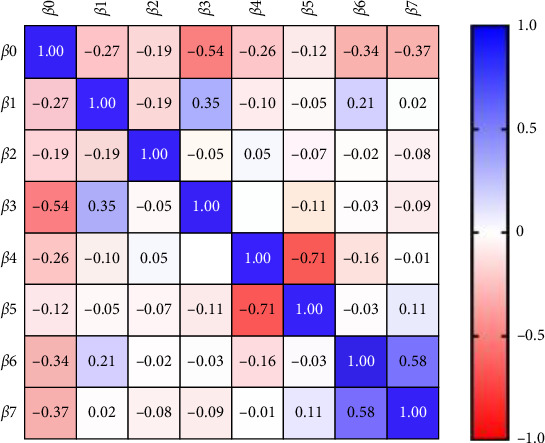
Parameter covariance in the third model of multiple linear regression.

Last, the third model of multiple linear regressions was done considering CAL as a dependent variable the result showed that there was a significant association with NGAL level in saliva as well as with other periodontal parameters and periodontal health status as presented in Table 7 and Figure 3.

## 4. Discussion

The main objective of the current investigation was to explore the association of periodontal health status with the salivary and serum levels of NGAL in patients with CKD with and without HD. The primary finding of this study reveals a rise in the prevalence of periodontitis among those with CKD, regardless of whether they were on HD or not. This finding is partially consistent with previous research that also revealed an elevated prevalence of periodontitis in patients undergoing HD [33]. Furthermore, a significant increase was found in the concentration of salivary NGAL in patients with CKD not on HD in comparison with patients on HD.

Subsequently, regression analysis revealed a significant association between salivary NGAL and clinical periodontal parameters as well as with patients who have CKD on HD. Conversely, CAL associated with salivary NGAL proposes the role of kidney disease in the progression of periodontal disease. Likewise, serum NGAL illustrated a significant association with CKD, whereas there was a nonsignificant association with periodontal health status along with clinical periodontal parameters as it was significantly detected at lower concentrations reduced in comparison with salivary concentration in the same group.

Performing direct evaluations of the existing literature with the current work is challenging primarily due to variations in study approach, sample characteristics, and measured outcomes. Significantly, the current study has assessed the periodontal health status of individuals with chronic renal disease, both with and without HD. In addition to the impact of biomarker levels on both diseases and vice-versa. The results of this investigation can be utilized to comprehend the coexistence of both illnesses, offering a fresh understanding of the underlying mechanisms that connect the two disorders, as well as discovering a noninvasive approach to monitor health conditions.

The increased serum and salivary NGAL levels in the CKD group were possibly due to several factors that stimulated renal tubular epithelial cells, leading to increased NGAL expression. NGAL can be absorbed by renal epithelial cells to regulate the expression of apoptosis-related proteins, which in turn promotes cell maturation and induces granulocyte apoptosis [34]. The subjects were experiencing CRF, leading to uremic syndrome, which has been related to immunological dysfunction, involving impairments in lymphocyte and monocyte activities [19]. However, the activation of toll-like receptor 4 in response to periodontal pathogens leads to an immune-inflammatory response that destroys tissue [35]. This response also induces the production of NGAL, which helps regulate inflammation and antimicrobial defense by affecting the function of neutrophils [36–41].

NGAL is a key biomarker of kidney injury and inflammation, commonly elevated in individuals with CKD and those undergoing dialysis. Its levels are influenced by factors such as acute infections, systemic inflammation, and oxidative stress. Understanding the impact of dialysis on NGAL levels, alongside inflammatory markers like C-reactive protein (CRP), could provide valuable insights into the systemic health challenges faced by CKD patients.

NGAL in CKD and Dialysis; NGAL is released during kidney injury and is often elevated in CKD patients. Dialysis itself can further influence NGAL levels, as the procedure is associated with increased oxidative stress and systemic inflammation. Studies have shown that NGAL levels rise during dialysis sessions due to these factors [42].

Relationship between NGAL and CRP; CRP is an acute-phase reactant that reflects systemic inflammation and is strongly associated with CKD progression and complications. Elevated CRP levels are common in CKD patients and are linked to a higher risk of cardiovascular events. NGAL, which is part of the innate immune response, often correlates with CRP levels, indicating the overall inflammatory burden in these patients [43].

Importance of assessing NGAL and CRP together by examining both NGAL and CRP levels provides a clearer understanding of the systemic inflammation in CKD patients. For instance, elevated levels of these markers are associated with increased periodontal tissue destruction and heightened systemic health risks [44]. This combined assessment could offer deeper insights into the interactions between kidney function, inflammation, and periodontal health.

NGAL is a multifunctional protein that enhances the stability of MMP-9 [45, 46], an enzyme that breaks down type IV collagen. This leads to an increased rate of degradation in the periodontium [47–49]. As a result, the strong inflammatory response leads to the rapid development of a secondary systemic inflammatory burden and the spread of periodontal pathogens and their locally generated substances such as lipopolysaccharides and cytokines throughout the body [50, 51]. NGAL expression can be stimulated by many cytokines and growth factors that may contribute to periodontal diseases, such as interleukin-1, IL-17, IL-22, insulin-like growth factor-1, transforming growth factor alpha, and tumor necrosis factor-alpha [46, 52, 53].

Clearing up its elevation and association with kidney and periodontal disease, as illustrated in the result of the present study, might explain the increased prevalence of periodontitis between patient groups. Besides, multiple studies have indicated that patients with severe periodontitis exhibit elevated amounts of NGAL in their saliva. This biomarker is mostly produced by neutrophils [17, 18, 54]. Therefore, NGAL may have a possible involvement in the development of periodontitis, similar to its established role in kidney disease [46, 48]. This is confirmed by the association of this biomarker in saliva with CAL in the second and third models of regression. Furthermore, NGAL concentration levels for the participants with gingivitis were elevated in participants included in the current study documenting the association of this biomarker with periodontal disease.

It is crucial to acknowledge that the current study has limitations. While the observed correlation is interesting, the study's cross-sectional nature prevents it from establishing a causative relationship. Longitudinal studies are needed to address causality. The cross-sectional design of our study was chosen because of the need for feasibility and decreased participant burden (many CKD patients have significant health problems). Additionally, as this study was conducted by a master's student, the researcher faced strict time limitations inherent to the academic timetable.

Hence, it is advisable to exercise caution while interpreting the data. Furthermore, the absence of healthy individuals for comparison and the absence of separate groups consisting solely of individuals with either periodontitis or chronic renal disease in a case-control study design may restrict the interpretation of certain findings, in addition to incorporating a larger sample size needed to enhance the generalizability of our findings. Finally, the duration of HD was not assessed in the present work, which would impact the periodontal health status. However, the study aimed to evaluate the association of the biomarker with periodontal health status and CKD which was replied with this study design. As a strength, the present study is the first that judged exclusively the association of serum and salivary NGAL with periodontal disease and CKD with and without HD.

## 5. Conclusion

In conclusion, based on the limitations of this study, the results indicate that salivary NGAL could serve as a biomarker linked to periodontal health in CKD patients undergoing HD. Additionally, there is a higher occurrence of periodontal disease among CKD patients who receive HD treatment. Nevertheless, additional research is required to investigate the specific mechanism via which periodontal diseases elevate the levels of this biomarker in saliva, as well as to assess its effectiveness in diagnosing or screening for periodontal diseases. These studies are necessary to establish this protein as a definitive biological marker of periodontal diseases. Furthermore, the existence of infectious and chronic inflammatory conditions, such as periodontal disease, might have a detrimental impact on the progression of chronic renal disease.

## Figures and Tables

**Table 1:** Periodontal health status for study groups.

PD	CKD without HD	CKD with HD	*p*-Value
	Number (%)	Number (%)	
Localized gingivitis^a^	8 (5.3)	3 (2)	0.12
Generalized gingivitis^a^	4 (2.6)	14 (9.3)	0.01 S
Localized periodontitis^a^	28 (18.6)	24 (16)	0.62
Generalized periodontitis^a^	35 (23.3)	34 (22.6)	0.72
Total gingivitis^a^	12 (8)	17 (11.3)	0.34
Total periodontitis^a^	63 (42)	58 (38.6)	0.32

*Note:* S = significant at (*p*  < 0.05).

Abbreviations: CKD, chronic kidney disease; HD, hemodialysis; PD, periodontal disease.

^a^Fisher exact test.

**Table 2:** Distribution of subjects according to age and sex between groups.

Periodontal disease	CKD without HD Mean ± SD age		CKD with HD Mean ± SD age		*p*-Value
Localized gingivitis	30 ± 8.159		29.333 ± 6.028		0.901
Generalized gingivitis	37.5 ± 6.028		38.933 ± 12.753		0.104
Total gingivitis	28.4 ± 5.45		36.09 ± 10.76		0.125
Localized periodontitis	44.8 ± 10.9		48.7 ± 10.7		0.187
Generalized periodontitis	50.30 ± 9.78		50.70 ± 6.9		0.842
Total periodontitis	48.30 ± 7.56		50.21 ± 6.96		0.752
Total age	46.30 ± 6.57		48.21 ± 6.78		0.340

**Sex distribution (*N*)**	**M**	**F**	**M**	**F**	

Localized gingivitis^a^	5	3	1	2	0.545
Generalized gingivitis^a^	4	0	8	7	0.245
Localized periodontitis^b^	19	19	12	12	0.259
Generalized periodontitis^b^	25	8	13	20	0.051
Total gingivitis^b^	9 (6%)	3 (2%)	9 (6%)	9 (6%)	0.17
Total periodontitis^b^	46 (30.6%)	17 (11.3%)	25 (16.6%)	32 (21.3%)	0.001*⁣*^*∗*^
Total sex	45 (30%)	30 (20%)	34 (22.6%)	41 (27.3%)	0.07

*Note:* Comparison for age was done using *t*-test. CKD with HD, chronic kidney disease with hemodialysis; CKD without HD, chronic kidney disease without hemodialysis.

Abbreviations: F, female; M, male; N, number; SD, standard deviation.

^a^Comparison by chi-square.

^b^Comparison by Fisher exact.

*⁣* ^*∗*^Significant at *p*-value < 0.01.

**Table 3:** Comparison of periodontal parameters between CKD patients with and without HD.

Parameters	Periodontal diseases (extent)	CKD without HD	CKD with HD	*p*-Value
		Mean ± SD	Mean ± SD	
PLI%	Localized gingivitis	30.37 ± 3.97	20 ± 3.6	<0.01***⁣*^*∗*^**
	Generalized gingivitis	30.53 ± 7.78	40.3 ± 9.78	>0.05
	Localized periodontitis	38.05 ± 7.96	43.58 ± 10.34	>0.05
	Generalized periodontitis	44.23 ± 6.56	41.59 ± 6.66	>0.05
	Total gingivitis	37.23 ± 12.63	31.42 ± 5.14	<0.05***⁣*^*∗*^**
	Total periodontitis	43.58 ± 8.36	42.37 ± 7.41	>0.05

BOP%	Localized gingivitis	18.85 ± 32.97	13.33 ± 1.156	<0.05***⁣*^*∗*^**
	Generalized gingivitis	41.55 ± 6.4	46.53 ± 5.25	>0.05
	Localized periodontitis	23.61 ± 5.12	26 ± 6.30	>0.05
	Generalized periodontitis	24.46 ± 4.69	24.97 ± 5.13	>0.05
	Total gingivitis	30.15 ± 4.16	29.93 ± 5.72	>0.05
	Total periodontitis	24.08 ± 4.87	25.40 ± 5.63	>0.05

PPD	Localized periodontitis	4.42 ± 1.48	4.96 ± 0.65	>0.05
	Generalized periodontitis	4.88 ± 1.70	5.6 ± 1.5	>0.05
	Total periodontitis	4.68 ± 1.23	5.32 ± 1.63	<0.05***⁣*^*∗*^**

CAL	Localized periodontitis	5.21 ± 1.93	5.71 ± 2.48	>0.05
	Generalized periodontitis	6.46 ± 2.14	6.74 ± 2.12	>0.05
	Total periodontitis	5.93 ± 2.13	6.29 ± 2.32	>0.05

*Note:* BOP%, percentage of bleeding on probing; CKD with HD, chronic kidney disease with hemodialysis; CKD without HD, chronic kidney disease without hemodialysis; PLI%, percentage of plaque index.

Abbreviations: CAL, clinical attachment loss; PPD, probing pocket depth; SD, standard deviation.

*⁣* ^*∗*^Level of significance at *p*-value < 0.05 (by *t*-test).

**Table 4:** NGAL levels in serum and saliva.

Biological fluid	Extent	CKD without HD	CKD with HD	*T*-test	*p*-Value
		Mean ( ± SD (ng/mL)	Mean ( ± SD (ng/mL)		
Serum	Localized gingivitis	225.533 (± 33.511)	319.817 (± 72.843)	3.074	0.013
	Generalized gingivitis	294.273 (± 97.354)	361.873 (± 78.196)	1.466	0.16
	Localized periodontitis	264.138 (± 78.08)	341.66 (± 99.451)	1.79	0.084
	Generalized periodontitis	266.811 (± 73.402)	286.078 (± 85.706)	0.668	0.508
	Total gingivitis	255.559 (± 72.06)	309.233 (± 75.838)	2.165	0.038
	Total periodontitis	269.628 (± 75.103)	320.764 (± 82.929)	1.289	0.202
	Total serum	274.138 (± 70.18)	351.66 (± 99.451)	1.85	0.090

Saliva	Localized gingivitis	521.955 (± 98.695)	395.167 (± 105.052)	1.87	0.04
	Generalized gingivitis	553.573 (± 84.089)	474.063 (± 110.479)	1.329	0.2
	Localized periodontitis	556.139 (± 106.633)	476.463 (± 105.337)	2.51	0.015
	Generalized periodontitis	537.395 (± 108.643)	449.483 (± 103.37)	3.207	0.002
	Total gingivitis	548.543 (± 108.18)	467.43 (± 123.944)	2.767	0.007
	Total periodontitis	539.054 (± 105.565)	457.164 (± 119.486)	3.348	0.001
	Total saliva	540.554 (± 100.595)	458.134 (± 99.450)	3.346	0.001
	Serum versus saliva	3.560		—	0.001

*Note*: CKD with HD, chronic kidney disease with hemodialysis; CKD without HD, chronic kidney disease without hemodialysis.

Abbreviation: SD, standard deviation.

**Table 5:** Association of serum levels of NGAL with periodontal health status and CKD.

Parameter estimates	Variable	Estimate	Standard error	95% CI (asymptotic)	|*t*|	*p*-Value
*β*0	Intercept	202.7	87.4	29.87 to 375.5	2.3	0.0*⁣*^*∗*^
*β*1	CKD [1]	63.0	27.1	9.526 to 116.6	2.3	0.0*⁣*^*∗*^
*β*2	NGAL saliva	0.0	0.1	−0.1914 to 0.2537	0.3	0.8
*β*3	PLI	−1.3	2.1	−5.513 to 2.955	0.6	0.6
*β*4	BOP	3.6	3.5	−3.326 to 10.48	1.0	0.3
*β*5	PPD	13.6	13.5	−13.15 to 40.39	1.0	0.3
*β*6	CAL	−10.7	9.2	−28.94 to 7.489	1.2	0.2
*β*7	Periodontal health [0]	13.6	47.3	−79.87 to 107.0	0.3	0.8

*Note:* The first model of multiple linear regression where NGAL in serum was the dependent variable. *R*^2^ = 0.06. BOP%, bleeding on probing percentage; PLI%, plaque index percentage.

Abbreviations: CAL, clinical attachment loss; CKD, chronic kidney disease; NGAL, neutrophil gelatinase-associated lipocalin; PPD, probing pocket depth; SD, standard deviation.

*⁣* ^*∗*^Level of significance at *p*-value < 0.05.

**Table 6:** Association of salivary levels of NGAL with periodontal health status and CKD.

Parameter estimates	Variable	Estimate	Standard error	95% CI (asymptotic)	|*t*|	*p*-Value
*β*0	Intercept	417.20	56.60	305.3 to 529.1	7.37	<0.0001
*β*1	CKD [1]	−82.84	19.42	−121.2 to −44.45	4.27	<0.0001
*β*2	NGAL serum	0.02	0.06	−0.1071 to 0.1419	0.28	0.78
*β*3	PLI%	0.08	1.60	−3.093 to 3.248	0.05	0.96
*β*4	BOP%	4.88	2.59	−0.2342 to 10.00	1.89	0.06
*β*5	PPD	22.20	9.99	2.446 to 41.95	2.22	0.03
*β*6	CAL	−18.83	6.74	−32.16 to −5.509	2.79	0.01
*β*7	Periodontal health [0]	−12.66	35.34	−82.52 to 57.20	0.36	0.72

*Note:* The second model of multiple linear regression where NGAL in saliva was the dependent variable. *R*^2^ = 0.19. BOP%, bleeding on probing percentage; PLI%, plaque index percentage.

Abbreviations: CAL, clinical attachment loss; NGAL, neutrophil gelatinase-associated lipocalin; PPD, probing pocket depth; SD, standard deviation.

**Table 7:** Association of CAL with biomarker and CKD.

Parameter estimates	Variable	Estimate	Standard error	95% CI (asymptotic)	|*t*|	*p*-Value
*β*0	Intercept	−0.092	0.810	−1.694 to 1.509	0.114	0.909
*β*1	CKD [1]	0.037	0.251	−0.458 to 0.533	0.148	0.883
*β*2	NGAL serum	−0.001	0.001	−0.0023 to 0.0006	1.164	0.246
*β*3	NGAL saliva	−0.003	0.001	−0.0047 to −0.0008	2.794	0.006
*β*4	PLI%	0.007	0.020	−0.03109 to 0.04599	0.382	0.703
*β*5	BOP%	0.088	0.031	0.02683 to 0.1494	2.842	0.005
*β*6	PPD	1.070	0.085	0.9031 to 1.238	12.650	<0.0001
*β*7	Periodontal health [0]	−2.402	0.380	−3.152 to −1.651	6.329	<0.0001

*Note:* The third model, *R*^2^ = 0.82. BOP%, bleeding on probing percentage; PLI%, plaque index percentage.

Abbreviations: CAL, clinical attachment loss; NGAL, neutrophil gelatinase-associated lipocalin; PPD, probing pocket depth; SD, standard deviation.

## Data Availability

Data will be made available upon request from the authors.
